# Statins Versus Proprotein Convertase Subtilisin/Kexin Type 9 (PCSK9) Inhibitors for Primary Cardiovascular Prevention in High-Risk Patients: A Systematic Review

**DOI:** 10.7759/cureus.83646

**Published:** 2025-05-07

**Authors:** Sangeen Khan, Ayesha Asif, Osman Omer, Muhammad Zubair Dawud Gondal, Hamza Tallal, Tajammul Abbas

**Affiliations:** 1 Internal Medicine, Fairfield General Hospital, Bury, GBR; 2 Internal Medicine, Karachi Medical and Dental College, Karachi, PAK; 3 Integrative Medicine, Prince Mohammed Bin Abdulaziz Hospital, Riyadh, SAU; 4 Surgery, Robina Mubashar Hospital, Mirpur, PAK; 5 Internal Medicine, Robina Mubashar Hospital, Mirpur, PAK; 6 Internal Medicine, Nishtar Medical University, Multan, PAK

**Keywords:** alirocumab, cardiovascular risk, evolocumab, hypercholesterolemia, ldl-c reduction, lipid-lowering therapy, pcsk9 inhibitors, primary prevention, randomized controlled trials, statins

## Abstract

Cardiovascular disease (CVD) remains a major global health concern, emphasizing the importance of effective primary prevention strategies, especially in individuals at high risk. While statins are foundational in lipid-lowering therapy, challenges such as inadequate low-density lipoprotein cholesterol (LDL-C) control or intolerance to high-dose statins persist. Proprotein convertase subtilisin/kexin type 9 (PCSK9) inhibitors have gained attention as promising agents in this context. This systematic review explores the comparative effectiveness of PCSK9 inhibitors versus traditional statin-based regimens in high-risk individuals without established CVD. The findings suggest that PCSK9 inhibitors offer robust LDL-C lowering capabilities and may improve goal attainment in primary prevention settings. Although direct evidence of cardiovascular event reduction is still emerging, early indications are favorable, particularly in genetically predisposed populations. Overall, PCSK9 inhibitors appear to be a viable adjunctive option for patients who do not achieve optimal lipid control with statins alone.

## Introduction and background

Cardiovascular disease (CVD) is the leading global cause of morbidity and mortality, responsible for approximately 18 million deaths annually [1]. Atherosclerotic cardiovascular disease (ASCVD), which includes coronary artery disease, peripheral arterial disease, and cerebrovascular disease, develops gradually and often remains asymptomatic until manifesting as myocardial infarction, stroke, or sudden cardiac death [2]. In response to this global burden, primary prevention strategies, targeting individuals at high risk before a first cardiovascular event, have become a central focus in preventive cardiology. High-risk individuals are commonly defined by the presence of multiple atherogenic risk factors (e.g., diabetes, familial hypercholesterolemia (FH), and chronic kidney disease), elevated low-density lipoprotein cholesterol (LDL-C) despite treatment, or a 10-year ASCVD risk ≥20% according to pooled cohort equations [3].

Among modifiable risk factors, dyslipidemia, particularly elevated LDL-C, is a key contributor to atherosclerosis [3]. Statins have long been the first-line therapy, with strong evidence supporting their LDL-C lowering and event-reducing effects across both primary and secondary prevention settings. However, limitations persist: up to 10-20% of patients experience statin intolerance, and many fail to reach guideline-recommended LDL-C targets even with high-intensity statins [4].

Proprotein convertase subtilisin/kexin type 9 (PCSK9) inhibitors, including alirocumab, evolocumab, and bococizumab, are monoclonal antibodies that reduce LDL-C by preventing PCSK9-mediated degradation of LDL receptors, thereby increasing hepatic clearance [5]. Originally approved for FH and secondary prevention, recent trials have evaluated their role in primary prevention among high-risk individuals inadequately controlled on statins alone [6]. These agents have demonstrated LDL-C reductions ranging from 36% to 68% in clinical trials [5,7]. Yet despite favorable efficacy, concerns remain regarding their high cost, potential long-term safety (e.g., neurocognitive effects), real-world adherence challenges, and limited cost-effectiveness in low-risk populations [8].

The review was guided by the PICO framework [9], which structured the research question as follows: the Population included adults at high risk for CVD without established ASCVD; the Intervention involved treatment with PCSK9 inhibitors such as alirocumab, evolocumab, or bococizumab; the Comparison was made against statin-based therapy or other standard lipid-lowering regimens; and the Outcomes assessed were threefold-efficacy, measured by LDL-C reduction and achievement of lipid targets; clinical outcomes, defined as the incidence of major cardiovascular events including myocardial infarction, stroke, or cardiovascular death; and safety, evaluated by the occurrence of adverse events, injection site reactions, and treatment discontinuation rates. In addition, this review acknowledges the critical role of social determinants, such as income, education, and access to healthcare, in shaping cardiovascular risk and influencing adherence to lipid-lowering therapies. These contextual factors are particularly important when considering the real-world application, accessibility, and cost-effectiveness of newer therapies like PCSK9 inhibitors.

## Review

Materials and methods

Search Strategy

The search strategy for this systematic review was developed in accordance with PRISMA (Preferred Reporting Items for Systematic Reviews and Meta-Analyses) guidelines [10] to ensure transparency and reproducibility. A comprehensive literature search was conducted across PubMed, Embase, ClinicalTrials.gov, and Cochrane CENTRAL to identify relevant randomized controlled trials comparing PCSK9 inhibitors with statin-based therapies in high-risk patients without established CVD. The search was restricted to English-language studies and clinical trials published within the last 10-12 years to ensure the inclusion of the most recent and high-quality evidence. The following keywords and subject headings (MeSH and Emtree terms) were used in various combinations: "PCSK9 inhibitors," "alirocumab," "evolocumab," "bococizumab," "statins," "HMG-CoA reductase inhibitors," "primary prevention," "cardiovascular disease," "high-risk patients," "randomized controlled trial," and "LDL-C reduction." Boolean operators such as AND, OR, and NOT were applied to optimize sensitivity and specificity. After removing duplicates, titles and abstracts were screened for relevance, followed by full-text assessment based on predefined PICO criteria. Only studies that enrolled high-risk primary prevention populations and reported lipid or cardiovascular outcomes were included in the final review.

Eligibility Criteria

The eligibility criteria for this systematic review were guided by a clearly defined PICO framework, which structured the inclusion parameters as follows: Population (P) - adult patients at high or very high risk of CVD, such as those with FH, diabetes, or significantly elevated LDL-C levels, but without a history of major cardiovascular events (e.g., myocardial infarction or stroke); Intervention (I) - treatment with PCSK9 inhibitors; Comparison (C) - statin-based therapies, including statins alone, dose escalation, or other lipid-lowering comparators such as ezetimibe; and Outcomes (O) - efficacy (LDL-C reduction and lipid goal attainment) and safety (adverse events or treatment discontinuation). Only randomized controlled trials that met these criteria, included participants on background statin therapy, and were published in English were included to ensure high methodological quality and relevance to the review question.

Trials were excluded if they focused exclusively on secondary prevention populations, lacked a direct comparator group involving statin therapy, or did not report relevant outcomes such as LDL-C reduction or cardiovascular events. Studies with mixed primary and secondary prevention populations were only included if subgroup data for primary prevention patients were extractable. Additionally, observational studies, narrative reviews, editorials, and trials without full-text availability were excluded. This stringent selection ensured that the included studies were both methodologically sound and directly applicable to the clinical question regarding the role of PCSK9 inhibitors in high-risk, statin-treated patients without established CVD.

Data Extraction

Data extraction was carried out systematically using a predefined template to ensure consistency and accuracy across all included studies. Key information extracted from each trial included the study title, authors, publication year, study design, population characteristics, type and dosage of PCSK9 inhibitor and comparator therapy, duration of follow-up, primary and secondary clinical or lipid-related outcomes, and relevant statistical measures such as percentage LDL-C reduction, hazard ratios, confidence intervals, and p-values. Special attention was given to identifying whether the study population represented a true primary prevention cohort and whether outcomes such as achievement of LDL-C targets and major adverse cardiovascular events were reported. All data were independently verified by multiple reviewers to minimize the risk of transcription errors or interpretive bias, and any discrepancies were resolved through discussion and consensus.

Data Analysis and Synthesis

Data analysis and synthesis were performed using a qualitative, narrative approach, as the heterogeneity in study designs, populations, dosing regimens, and outcome reporting precluded a formal meta-analysis. The findings from each included randomized controlled trial were systematically reviewed and compared in terms of LDL-C reduction, achievement of lipid targets, and, where available, cardiovascular event outcomes. Trials were grouped based on the type of PCSK9 inhibitor used and their respective comparators, with emphasis placed on studies involving high-risk primary prevention populations. Key patterns, such as consistency in lipid-lowering efficacy and safety profiles, were identified and summarized to highlight the comparative effectiveness of PCSK9 inhibitors versus statin-based strategies. In addition, attention was given to subgroup trends, such as treatment response in FH or diabetic patients, and treatment-emergent adverse events were analyzed descriptively to assess tolerability across interventions.

Results

Study Selection Process

**Figure 1:**
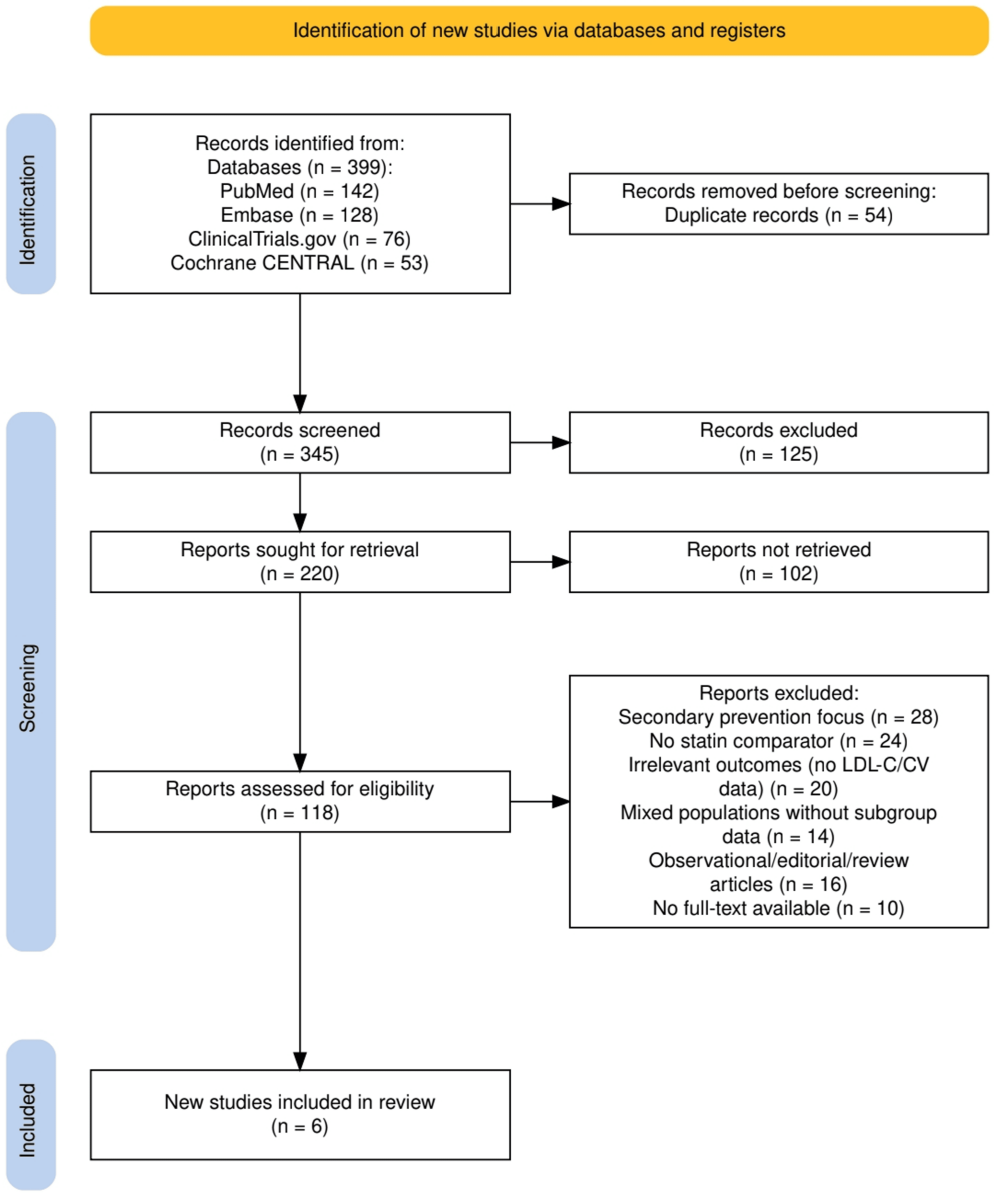
The study selection process depicted by the PRISMA flowchart. PRISMA: Preferred Reporting Items for Systematic Reviews and Meta-Analyses

The study selection process, as given in Figure 1, was conducted in alignment with PRISMA guidelines to ensure methodological rigor and transparency. A total of 399 records were identified through database searches, including PubMed (142), Embase (128), ClinicalTrials.gov (76), and Cochrane CENTRAL (53). After removing 54 duplicate entries, 345 records were screened based on titles and abstracts. Of these, 125 were excluded for not meeting the initial relevance criteria. The remaining 220 full-text reports were sought for retrieval, of which 102 could not be accessed. The remaining 118 full-text articles were assessed for eligibility. A total of 112 studies were excluded for reasons including secondary prevention focus (n=28), lack of a statin comparator (n=24), irrelevant outcomes (n=20), mixed populations without subgroup data (n=14), study type (observational/editorial/review) (n=16), and unavailability of full text (n=10). Ultimately, six studies met the inclusion criteria and were included in the final systematic review.

Characteristics of the Selected Studies

The characteristics of the six studies included in this systematic review are summarized in Table 1. All selected studies were randomized controlled trials with varying designs, including double-blind, placebo-controlled, active-controlled, and open-label formats. Study populations consisted of high or very high cardiovascular risk patients without established CVD, with LDL-C thresholds ranging from ≥70 to ≥100 mg/dL, and all participants were on background statin therapy. The interventions evaluated different PCSK9 inhibitors, lerodalcibep, alirocumab, evolocumab, and bococizumab, administered either biweekly or monthly, and were compared to either placebo, ezetimibe, intensified statin regimens, or alternative lipid-lowering strategies. Follow-up durations ranged from 12 to 52 weeks. Primary outcomes across trials primarily focused on percent change in LDL-C, while one trial also reported major adverse cardiovascular events. Across studies, PCSK9 inhibitors consistently achieved LDL-C reductions between 36.3% and 68.6%, significantly outperforming comparator therapies. Statistical analyses demonstrated strong significance in lipid-lowering efficacy (p<0.001 in most studies), with some reporting additional clinical event data, such as a hazard ratio of 0.83 in an FH subgroup for cardiovascular outcomes.

**Table 1:** The summary of the characteristics of the selected studies. LDL-C, low-density lipoprotein cholesterol; CVD, cardiovascular disease; mITT: modified intention-to-treat; AEs, adverse events; SC, subcutaneous; Q2W: every 2 weeks (biweekly); QM: every month (monthly); MI: myocardial infarction; HR: hazard ratio; CI: confidence interval; SE: standard error; CV: cardiovascular; FH: familial hypercholesterolemia

Study (Author, Year)	Study Design	Population Characteristics	Intervention (PCSK9 Inhibitor)	Comparator (Statin/Other)	Follow-Up Duration	Primary Outcomes	Key Findings/Results	Statistical Data (HR, CI, p-values)
Klug et al., 2024 (LIBERATE-HR) [11]	Randomized, double-blind, placebo-controlled phase 3 trial	922 participants, mean age 64.5 years, LDL-C ≥70 mg/dL (CVD) or ≥100 mg/dL (high risk), all on maximally tolerated statins	Lerodalcibep 300 mg monthly, subcutaneous	Placebo (all participants on statins)	52 weeks	Percent change in LDL-C at week 52 and average of weeks 50-52	Lerodalcibep reduced LDL-C by 56.2% (mITT); 90% achieved ≥50% LDL-C reduction and target levels	LDL-C reduction: 56.2% (SE 2.2%) at week 52, p<0.001; AEs similar except more injection site reactions
Cannon et al., 2015 (ODYSSEY COMBO II) [12]	Randomized, double-blind, double-dummy, active-controlled trial	720 high-CV-risk patients with inadequately controlled LDL-C despite maximally tolerated statins	Alirocumab 75 mg SC every 2 weeks + oral placebo	Ezetimibe 10 mg daily + SC placebo	52 weeks (interim analysis)	Percent reduction in LDL-C from baseline, proportion achieving LDL-C <1.8 mmol/L	Alirocumab reduced LDL-C by 50.6% vs. 20.7% with ezetimibe; 77% vs. 45.6% reached LDL-C <1.8 mmol/L	Difference in LDL-C reduction: 29.8±2.3%, p<0.0001; LDL-C: 1.3 vs. 2.1 mmol/L at week 24
Bays et al., 2015 (ODYSSEY OPTIONS I) [13]	Randomized, multicenter, open-label, parallel-group trial with blinded dose escalation	355 high/very high-risk patients, LDL-C ≥70 or ≥100 mg/dL on atorvastatin 20/40 mg	Alirocumab 75 mg Q2W (increased to 150 mg Q2W if needed) + atorvastatin	Ezetimibe add-on, doubling atorvastatin dose, or switching to rosuvastatin 40 mg	24 weeks	Percent change in LDL-C from baseline to 24 weeks	LDL-C reduced 44.1% (atorva 20 mg) & 54.0% (atorva 40 mg) vs. 20.5%-22.6% (ezetimibe), 5.0%-4.8% (double statin), 21.4% (rosuva)	Alirocumab vs. all comparators: p<0.001; goal achievement: 87.2% and 84.6%
Farnier et al., 2016 (ODYSSEY OPTIONS II) [14]	Randomized, multicenter, open-label, parallel-group trial with blinded dose escalation	305 patients with CVD or risk factors, LDL-C ≥70 mg/dL (CVD) or ≥100 mg/dL (risk factors), on rosuvastatin 10/20 mg	Alirocumab 75 mg Q2W (up to 150 mg Q2W if needed) + rosuvastatin	Add-on ezetimibe 10 mg/day or doubling rosuvastatin dose	24 weeks	Percent change in LDL-C from baseline to 24 weeks	LDL-C reduced by 50.6% (rosuva 10 mg) and 36.3% (rosuva 20 mg) vs. 14.4%-11% (ezetimibe), 16.3%-15.9% (double statin)	p<0.0001 vs. comparators (rosuva 10 mg); p=0.0136/0.0453 (rosuva 20 mg); 84.9%-66.7% reached targets
Hirayama et al., 2014 (YUKAWA Study) [15]	Randomized, double-blind, placebo-controlled, phase 2 trial	310 Japanese high-CV-risk patients on stable statin ± ezetimibe; mean age 62	Evolocumab 70/140 mg Q2W or 280/420 mg QM	Placebo (all on background statins)	12 weeks	Percent change in LDL-C from baseline (ultracentrifugation method)	LDL-C reduction: -68.6% (140 mg Q2W), -63.9% (420 mg QM); up to 96% reached LDL-C <1.8 mmol/L	LDL-C reduction vs. placebo: -68.6% (SE 3.0), -63.9% (SE 3.2); AE rate: 51% vs. 38%
Ridker et al., 2018 (SPIRE Trials – FH Subgroup) [16]	Randomized, placebo-controlled, multicenter trials (pooled SPIRE analysis)	1578 FH patients on statins; 42% primary prevention; mean age 58, baseline LDL-C >100 mg/dL	Bococizumab 150 mg SC every 2 weeks	Placebo (all on statins)	Median 11.2 months	Major adverse cardiovascular events (MI, stroke, CV death)	CV events: 18/781 (bococizumab) vs. 22/797 (placebo); LDL-C reduced 55%; similar efficacy to non-FH group	HR=0.83; 95% CI: 0.44-1.54; p=0.55 (FH group); non-FH: HR=0.79; CI: 0.64-0.97; p=0.023

Quality Assessment

The quality assessment of the included studies, as detailed in Table 2, was conducted using the Cochrane Risk of Bias 2.0 [17] tool for randomized controlled trials and the ROBINS-I tool for the post-hoc subgroup analysis. Overall, the methodological quality of the studies was high to moderate. Three trials LIBERATE-HR [11], ODYSSEY COMBO II [12], and YUKAWA [15] were rated as high quality, with low risk of bias across all domains, including selection, performance, detection, attrition, and reporting. Two studies ODYSSEY OPTIONS I [13] and II [14] were rated as having moderate quality, primarily due to open-label designs introducing potential performance bias. The SPIRE trial subgroup [16] analysis, evaluated using ROBINS-I [18], was also assigned a moderate quality rating due to its post-hoc nature, with moderate risk in selection and performance bias, and some concerns regarding reporting. Despite these limitations, all included trials maintained methodological rigor sufficient to support reliable interpretation of their findings.

**Table 2:** A representation of the quality assessment of each of the selected studies. RCT: randomized controlled trial; RoB 2.0: Risk of Bias Version 2.0 (Cochrane tool for RCTs); ROBINS-I: Risk Of Bias In Non-randomised Studies - of Interventions; FH: familial hypercholesterolemia

Study (Author, Year)	Tool Used	Study Design	Selection Bias	Performance Bias	Detection Bias	Attrition Bias	Reporting Bias	Overall Risk of Bias
Klug et al., 2024 (LIBERATE-HR) [11]	Cochrane RoB 2.0	Phase 3 RCT	Low	Low	Low	Low	Low	Low Risk of Bias
Cannon et al., 2015 (ODYSSEY COMBO II) [12]	Cochrane RoB 2.0	RCT	Low	Low	Low	Low	Low	Low Risk of Bias
Bays et al., 2015 (ODYSSEY OPTIONS I) [13]	Cochrane RoB 2.0	RCT (open-label)	Low	Some concerns (open-label; may affect adherence or reporting)	Low	Low	Low	Some Concerns
Farnier et al., 2016 (ODYSSEY OPTIONS II) [14]	Cochrane RoB 2.0	RCT (open-label)	Low	Some concerns (open-label; may affect adherence or reporting)	Low	Low	Low	Some Concerns
Hirayama et al., 2014 (YUKAWA Study) [15]	Cochrane RoB 2.0	Phase 2 RCT	Low	Low	Low	Low	Low	Low Risk of Bias
Ridker et al., 2018 (SPIRE Trials – FH) [16]	ROBINS-I	Post-hoc subgroup of pooled RCTs (non-randomized analysis)	Moderate	Moderate (post-hoc nature)	Low	Low	Some concerns	Moderate Risk of Bias

Discussion

In this systematic review of six randomized clinical trials involving patients at high cardiovascular risk but without established CVD, PCSK9 inhibitors consistently demonstrated superior efficacy in lowering LDL-C levels compared to traditional statin-based therapies or adjunctive lipid-lowering strategies. Across multiple studies, the addition of PCSK9 inhibitors such as alirocumab, evolocumab, lerodalcibep, and bococizumab to background statin therapy resulted in LDL-C reductions ranging from 36.3% to 68.6%, significantly outperforming comparators like ezetimibe, statin dose escalation, or placebo. For instance, in the ODYSSEY COMBO II trial [12], alirocumab achieved a 50.6% LDL-C reduction versus 20.7% with ezetimibe (p<0.0001), while the YUKAWA trial [15] reported reductions up to 68.6% with evolocumab. Additionally, the LIBERATE-HR trial [11] demonstrated that 90% of patients treated with lerodalcibep achieved both a ≥50% reduction in LDL-C and target lipid levels at 52 weeks, compared to a notably lower proportion in the placebo group.

Beyond lipid control, the cardiovascular outcome data, though more limited, also support a potential advantage of PCSK9 inhibitors in high-risk primary prevention populations. The SPIRE subgroup analysis [16] involving 1,578 patients with FH reported a hazard ratio of 0.83 (95% CI: 0.44-1.54) for major cardiovascular events with bococizumab compared to placebo, reflecting a trend toward benefit despite the short follow-up and lack of statistical significance (p=0.55). In the broader non-FH population within the same trial, a significant reduction in events was observed (HR=0.79; 95% CI: 0.64-0.97; p=0.023), suggesting consistency in effect. While event-based data remain relatively sparse in primary prevention cohorts, the robust lipid-lowering effect, high rates of target achievement, and acceptable safety profiles collectively highlight the potential of PCSK9 inhibitors as a powerful option in high-risk patients who are inadequately managed with statins alone.

While this review highlights the promising lipid-lowering efficacy of PCSK9 inhibitors, the evidence for cardiovascular event reduction in true primary prevention settings remains limited. Notably, the SPIRE subgroup analysis suggested a potential trend toward benefit (HR=0.83), but this was not statistically significant (95% CI: 0.44-1.54; p=0.55), and such findings should be interpreted with caution. Trends must be distinguished from statistically robust outcomes to avoid overstatement. Across trials, adverse events were generally mild, with injection site reactions (ranging from 4% to 7%) being the most frequent. Serious events and treatment discontinuations were rare. However, cost remains a major barrier: as of 2024, annual treatment with PCSK9 inhibitors may range from $4,500 to $6,000 USD in high-income countries, while access in low- and middle-income countries is extremely limited. Furthermore, the restrictive inclusion criteria in trials, favoring patients with high adherence potential and fewer comorbidities, may limit applicability to broader real-world high-risk populations. Future research must focus on long-term outcomes, cost-effectiveness, and real-world effectiveness in diverse, primary prevention populations to fully define the role of PCSK9 inhibitors. The findings of this review align with and extend current evidence presented in lipid management guidelines by both the European Society of Cardiology (ESC) [19] and the American College of Cardiology/American Heart Association (ACC/AHA), which emphasize aggressive LDL-C reduction in high-risk patients, including those without manifest ASCVD [20]. Both guidelines endorse the use of PCSK9 inhibitors in individuals at very high risk who fail to meet lipid goals on maximally tolerated statin therapy. Previous studies, such as those by Navarese et al. [21] and Schmidt et al. [22], have similarly demonstrated the lipid-lowering efficacy and cardiovascular event reduction potential of PCSK9 inhibitors, although many of those included predominantly secondary prevention cohorts. This review contributes uniquely by focusing exclusively on the primary prevention setting, reinforcing that PCSK9 inhibitors achieve significant LDL-C reductions and suggesting a possible extrapolation of cardiovascular benefits even in patients without previous events, thereby expanding the scope of existing evidence. The real-world implications of these findings are particularly relevant for a growing population of patients with elevated cardiovascular risk who do not yet exhibit overt disease but struggle to reach guideline-recommended LDL-C targets using statins alone. The consistent and significant LDL-C reductions observed with PCSK9 inhibitors in this review, often exceeding 50%, and with up to 90% of patients meeting target goals in some trials, highlight the potential for these agents to bridge therapeutic gaps in high-risk populations, such as those with FH, diabetes, or statin intolerance [23]. Achieving and maintaining lower LDL-C levels has a well-established correlation with long-term reductions in cardiovascular morbidity and mortality, as emphasized by landmark trials like FOURIER and ODYSSEY OUTCOMES. While those trials were largely in secondary prevention settings, this review supports the notion that early intensive lipid management in primary prevention could yield similar long-term advantages, justifying the selective use of PCSK9 inhibitors where appropriate.

Despite their proven efficacy, the widespread use of PCSK9 inhibitors is hindered by significant cost-related barriers, particularly in low- and middle-income countries where healthcare resources are limited and access to specialty medications remains constrained [24]. As a result, statins remain the first-line therapy for primary prevention due to their affordability, extensive evidence base, and overall tolerability. However, PCSK9 inhibitors may be justified in select high-risk patients who are either statin-intolerant or unable to achieve LDL-C targets despite maximal statin therapy, especially those with FH or diabetes [25]. In terms of safety, the included trials generally reported a favorable profile for PCSK9 inhibitors, with adverse events comparable to placebo or standard therapy. The most commonly reported issue was mild-to-moderate injection site reactions, observed in up to 6.9% of patients receiving lerodalcibep [11], while sporadic antidrug antibody formation was noted in trials like SPIRE and LIBERATE-HR, though these did not significantly impact efficacy or lead to treatment discontinuation. Overall, the safety data support the tolerability of PCSK9 inhibitors, reinforcing their potential role in targeted, high-need populations.

This systematic review has several notable strengths, including the exclusive inclusion of high-quality randomized controlled trials with low to moderate risk of bias, and a specific focus on primary prevention in high-risk patients, a subgroup often underrepresented in cardiovascular outcome studies. The inclusion of recent trials such as LIBERATE-HR ensures the synthesis reflects the latest advancements in lipid-lowering therapy, and the structured use of a PICO-based selection strategy enhances the review’s clinical relevance. However, some limitations must be acknowledged. The total number of eligible studies remains relatively small- and long-term cardiovascular outcome data are limited, particularly in purely primary prevention cohorts. Additionally, the review did not perform a meta-analysis, which may limit the quantitative synthesis of effect sizes across trials. Potential publication bias and the heterogeneity in interventions (different PCSK9 molecules and dosing regimens) further constrain generalizability, underscoring the need for more dedicated, long-duration studies focusing on clinical outcomes in high-risk, event-free populations.

Future research should prioritize large-scale, long-term randomized controlled trials specifically designed to assess the efficacy of PCSK9 inhibitors in primary prevention populations, particularly those with multiple risk factors but no established CVD. While current evidence demonstrates robust LDL-C reductions, data on long-term cardiovascular event reduction in purely primary prevention cohorts remain limited. Head-to-head studies comparing PCSK9 inhibitors with intensive statin therapy or combination regimens, including cost-effectiveness analyses, are essential to guide resource allocation, especially in low- and middle-income settings. Additionally, real-world evidence on adherence, patient-reported outcomes, and healthcare system impact will be crucial in defining the optimal positioning of PCSK9 inhibitors within preventive cardiology.

## Conclusions

This systematic review highlights the substantial LDL-C lowering efficacy and acceptable safety profile of PCSK9 inhibitors in high-risk patients without established CVD who remain inadequately controlled on statins alone. While statins remain the cornerstone of primary prevention due to their affordability and robust evidence base, PCSK9 inhibitors may serve as a valuable adjunct for selected patients, particularly those with familial hypercholesterolemia, type 2 diabetes, or elevated lipoprotein(a), who are unable to achieve guideline-recommended lipid targets or are statin-intolerant. Our review is among the few to focus specifically on high-risk individuals in primary prevention settings, offering a targeted synthesis distinct from prior studies centered on secondary prevention. However, most long-term cardiovascular outcome data still derive from secondary prevention trials or mixed-population analyses, underscoring the need for future research that directly evaluates clinical endpoints in purely primary prevention cohorts. Until such data emerge, PCSK9 inhibitors should be considered a personalized option in clearly defined high-risk groups where traditional therapies prove insufficient.
